# The Efficacy of Imiquimod-Induced Psoriasis Model on Murine Cells

**DOI:** 10.7759/cureus.62914

**Published:** 2024-06-22

**Authors:** Tony Joseph, Mark Genkin, Alexander Genkin, John Joseph, Eddy Manuchian, Kathryn Ray

**Affiliations:** 1 Department of Biology, City University of New York-Brooklyn College, Brooklyn, USA; 2 Department of Biology, City University of New York-Macaulay Honors College, Brooklyn, USA; 3 Department of Biology, Stuyvesant High School, Brooklyn, USA; 4 Department of Biology, Montgomery Blair High School, Silver Spring, USA; 5 Department of Rheumatology, Veteran Affairs Medical Center, Brooklyn, USA; 6 Department of Microbiology, City University of New York-Brooklyn College, Brooklyn, USA

**Keywords:** keratinocyte, imiquimod, model, psoriasis, imq

## Abstract

Keratinocytes are an essential component of the epidermis that undergoes constant proliferation and differentiation. However, the dysregulation of keratinocyte differentiation has been implicated in various skin disorders such as psoriasis. Imiquimod, otherwise known as IMQ, is a topical immunomodulator often used to induce psoriasis-like lesions in murine models for research purposes. This study focuses on the efficacy of using IMQ to induce a psoriasis-like model on murine skin cells by analyzing single-cell RNA sequencing and trajectory analysis. The results indicate a few differences between IMQ-induced and control murine cells, primarily the increased keratinocyte and immune cell populations, which reflects the cell identity found on psoriatic skin. However, trajectory analysis reveals that IMQ-induced cells have quite a linear differentiation pattern compared to the branched pattern found in control cells. As a result, further research must be conducted to explore differing factors between psoriatic cells and IMQ-induced cells to determine its usefulness in mimicking psoriasis-like conditions for research.

## Introduction

The epidermis is the outermost layer of skin and is essential in protecting the body from pathogens and chemical contamination [1,2]. Keratinocytes are found in multiple epidermis layers, forming fibrous keratin proteins and granules in their cytoplasm. In their cornified layer, they die without their nuclei or organelles before being completely removed by desquamation [3]. The keratinocytes undergo differentiation by various signaling pathways, transcription factors, and microenvironmental cues [4]. Any disruptions in this process can lead to skin disorders or impaired barrier function [5].

There are many other cell types found in the epidermis, some of which include sebocytes (sebaceous gland cells), inner bulge keratinocytes, outer bulge keratinocytes, differentiated suprabasal keratinocyte cells, terminally differentiated keratinized layer cells, and upper hair follicle cells. Sebocytes are differentiated epithelial cells within sebaceous glands that accumulate lipids and release sebum through holocrine secretion [6]. Inner bulge keratinocytes are in the inner bulge region of hair follicles, and maintain the regeneration of hair follicles [7]. Outer bulge keratinocytes are on the outer portion of the follicle and support the stem cells to participate in the repair and regeneration of the skin and hair [7]. Differentiated cells undergo specialization to perform protective skin barriers of hair structures [8]. Terminal differentiated keratinized layer cells are matured keratinocytes that form a tough outer skin layer called the stratum corneum, which acts as a barrier [8]. Finally, the upper hair follicle cells are on the upper part of the hair follicle and have differentiated keratinocytes that contribute to the structure of the hair shaft and surrounding epidermis [7].

Epidermal keratinocytes have a high capacity for proliferation by stem cell replication in the basal layer, making them useful in vitro experiments for the study of wound healing, drug screening, and cosmetic/antiaging products [9]. Its property of senescence is also important in understanding causes of skin aging like β-galactosidase activity, telomere shortening, and the expression of secretory phenotype factors and how it causes psoriasis, eczema, skin cancer, and genetic disorders such as epidermolysis bullosa [9].

Psoriasis is a chronic inflammatory skin disease with significant autoimmune involvement [10]. There are two main forces driving the buildup of cells on the surface of the epidermis in psoriasis: keratinocyte proliferation and immune cell infiltration. At the cellular level of an individual with psoriasis, immune cells are known to secrete heterodimeric cytokine interleukin (IL) 23 [11]. The function of IL-23 is to initiate an inflammatory response, which results in the increase in T helper 17 (Th17) cells. Th17 cells contribute to an increase in the production of keratinocytes [12]. Keratinocyte activation results in keratinocyte proliferation, which results in epidermal hyperplasia or psoriasis.

Imiquimod (IMQ) is a skin treatment medication administered as a cream to the affected area [13]. IMQ is a topical immunomodulator that influences the immune response by affecting the functions of skin immune cells, such as T lymphocytes [13].

Psoriasis is often induced by IMQ to understand the pathogenesis of psoriasis and evaluate potential therapeutic treatments [13]. IMQ can be applied to the skin so that toll-like receptors (TLRs) can produce pro-inflammatory cytokines and chemokines in increasing keratinocyte proliferation and the production of immune cells [13]. The local inflammation allows the immune cells to invade the skin, further amplify the inflammation, and create psoriasis-like lesions. IMQ acts on the pathway of toll-like receptor 7 (TLR7) and toll-like receptor 8 (TLR8), pattern recognition receptors expressed on immune cells, which are necessary in the recruitment of adaptor proteins such as myeloid differentiation primary response 88 (MyD88) and the activation of downstream signaling molecules, including interleukin-1 receptor-associated kinase (IRAK) and tumor necrosis factor receptor-associated factor 6 (TRAF6) [14]. This cascade of signals ultimately activates transcription factors, such as nuclear factor kappa-light-chain-enhancer of activated B cells (NF-κB) and interferon regulatory factor (IRF), to cause the release of the previously mentioned cytokines and chemokines vital to the reaction [14].

However, the IMQ-induced psoriasis model fails to capture the full scope of the psoriasis pathogenesis, including genetic predisposition, immune dysregulation, environmental factors, and skin barrier dysfunction [15]. In addition, although IMQ is efficient in imitating plaque psoriasis, it may not represent other subtypes such as guttate psoriasis and pustular psoriasis accurately [16]. Differences in immune responses indicate that the results are skewed in the efficacy of certain therapies and that we need to validate findings from the IMQ model in human clinical studies to ensure their translational relevance [16].

## Materials and methods

Epidermal cells for this study were isolated from adult mice by Greenberg et al. in their research that determines the effects of circadian control of interferon-sensitive gene expression in murine skin [17]. In their study, experimental mice had 1% IMQ topical treatment applied to their skin over the course of five days. Inflammatory markers were observed to be significantly elevated after five days of topical treatment of 1% IMQ, in comparison to wild-type mouse.

The single-cell RNA data we used in our study was obtained from series GSE142345 on Gene Expression Omnibus (GEO). The two provided samples, one consisting of single-cell RNA sequencing data of the skin from control mice that were shaved and the other consisting of single-cell RNA sequencing data of the skin from mice that were shaved and treated with 1% IMQ for six hours, were downloaded and analyzed using the R software (R Foundation for Statistical Computing, Vienna, Austria).

After cell sorting, Seurat, an R package, (Satija Lab, New York City, NY) was used to analyze the single-cell RNA sequences through clustering methods.

Quality control was performed on both the control and IMQ datasets using Seurat 4.0 to control for the number of features (0 < nFeature_RNA < 4500), as well as the percentage of mitochondrial DNA (<10%). Three thousand five hundred ninety-five remaining cells in the control group and 1679 remaining cells in the IMQ group were further analyzed. The variance-stabilizing transformation (VST) selection method was used to find the top 2000 variable features. Principal component analysis was performed using the JackStraw method. Clusters were found using the FindClusters method. Resolution 1.5 and 2.0 were used, respectively, for the control and IMQ groups. Twenty-one clusters were found in both groups.

The identification of cluster cell types was done visually, using VlnPlot and FeaturePlot (uniform manifold approximation and projection {UMAP}), as well as previously identified characteristic marker genes (Table 1), derived from Joost et al. [18]. Twelve cell types were identified in the control group, and 10 cell types were identified in the IMQ group.

**Table 1:** Gene Expression Markers Associated With Corresponding Cell Type

Cell Type	Gene Marker
Sebocytes (Sebaceous Gland Cells)	Scd1/Mgst1
Inner Bulge Keratinocytes	Krt6a/Krt75
Outer Bulge Keratinocytes	Cd34/Postn
Differentiated Suprabasal Keratinocyte Cells	Krt10/Ptgs1
Terminally Differentiated Keratinized Layer Cells	Lor/Flg2
Upper Hair Follicle Cells	Krt79/Krt17
Langerhans Cells	Cd207^+^/Ctss^+^
Resident T Cells	Cd3^+^/Thy1^+^

Dimension reduction is used to develop t-distributed stochastic neighbor embedding (tSNE) and UMAP plots such that a plot representing all the cells in the sample, and their similarities to each other, is possible. Seurat first plots the cells in multidimensional space, with each dimension representing a different feature being analyzed. Then, this plotting is flattened into two-dimensional space, preserving the distance between genetically distinct cells.

Seurat is also used to develop monocle trajectories, graphs that use the notion of pseudotime, which is a measure of biological progression such as cell differentiation. Monocle 2.20 (Cole Trapnell’s lab, Seattle, WA) was used to project differentiation trajectories of control and IMQ keratinocytes. Trajectories were first marked with previously identified cell types and then by marker genes indicative of keratinocyte proliferation and differentiation [19]. The markers Krt1, Krt10, Krt14, Krt5, and Mki67 were looked at, with a specific focus on Krt1 and Krt10 gene markers.

## Results

Seurat was used to identify cell cluster groups based on gene expression data and t-distributed stochastic neighbor embedding (tSNE) in order to arrange the cells in groups, which can then be identified using Table 1.

**Figure 1:**
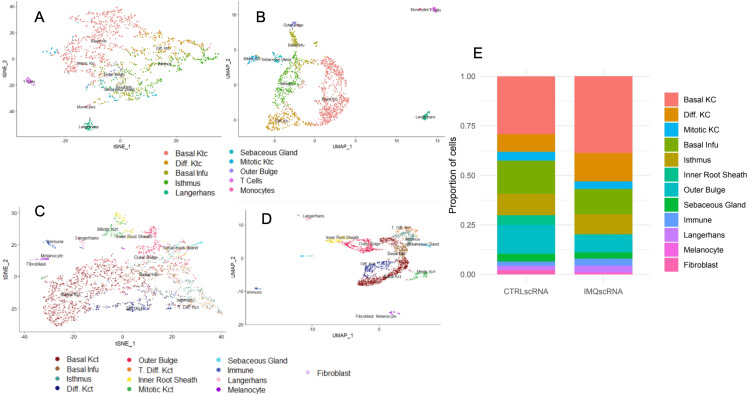
Epidermal Cell Populations of Murine Cells From Control and Psoriatic Groups (A) tSNE and UMAP dimensional reduction plots of 1% IMQ-applied murine cells. (B) UMAP dimensional reduction plots of 1% IMQ-applied murine cells. (C) tSNE and UMAP dimensional reduction plots of control murine cells. (D) UMAP dimensional reduction plots of control murine cells. (E) Cell identity comparison between 1% IMQ-applied murine cells and control cells tSNE, t-distributed stochastic neighbor embedding; UMAP, uniform manifold approximation and projection; IMQ, imiquimod; KC, keratinocyte cell; CTRL, control; scRNA, small conditional RNA

Data from single-cell RNA sequencing on 1% IMQ-applied murine cells resulted in 1679 cells after data cleaning. After filtering the results with a 2.0 resolution, 21 clusters were refigured as 10 clusters, including basal keratinocytes, differentiated keratinocytes, basal infundibulum cells, isthmus cells, Langerhans cells, sebaceous gland cells, mitotic keratinocytes, outer bulge keratinocytes, T cells, and monocytes (Figure 1A and Figure 1B).

Data from single-cell RNA sequencing on control murine cells resulted in 3595 cells after data cleaning. After filtering the results with a 1.5 resolution, 21 clusters were refigured in 12 clusters, including basal keratinocytes, basal infundibulum cells, isthmus cells, differentiated keratinocytes, outer bulge keratinocytes, terminally differentiated keratinocytes, inner root sheath cells, mitotic keratinocytes, sebaceous gland cells, immune cells, Langerhans cells, melanocytes, and fibroblasts (Figure 1C and Figure 1D).

The majority of identified cell types were found to be present within both the control and IMQ datasets. The identified cell types common to both datasets included basal keratinocytes, differentiated keratinocytes, mitotic keratinocytes, Langerhans cells, isthmus cells, basal infundibulum cells, outer bulge cells, and sebaceous gland cells. T cells and monocytes were indistinguishable in the control dataset and were grouped into an immune cell type. However, they were distinguishable in the IMQ dataset and kept in their separate categories. Inner root sheath cells, melanocytes, and fibroblasts were found only in the control dataset; they were absent in the IMQ dataset.

Across both tSNE and UMAP plots, cell types were more widespread in IMQ samples compared to control samples, revealing the diversity in heterogenous cell identity, which was also indicated by previous research [20] that used different gene markers for each cell type.

Differences in cell proportions are quite vast between IMQ-induced and control murine cells (Figure 1E). By looking at the clusters that were similar in the control and IMQ cell clusters, the percent composition of cells can be examined. The IMQ-treated cells contain a significant increase in keratinocytes, both basal and differentiated, and immune cells in comparison to the control cells (Figure 1E). In addition, the IMQ-induced murine cells contained less hair follicle cells (basal infundibulum, isthmus, and inner root sheath cells) when compared to control murine cells (Figure 1E).

Across other studies, many different resolutions were used to identify cell cluster groups, with the most used being a 0.5 resolution [20,21]. However, when implementing a 0.5-resolution tSNE plot with the single-cell RNA sequencing data, too few cell clusters were identified as unique, resulting in the loss of many cell groups. As a result, higher-resolution tSNE plots were needed for this study.

To better understand the differentiation patterns of IMQ and control keratinocytes, Monocle was used to plot pseudotime trajectories that order the keratinocytes in terms of their transition states. Two cell states were found, specifically basal keratinocytes and differentiated keratinocytes.

**Figure 2:**
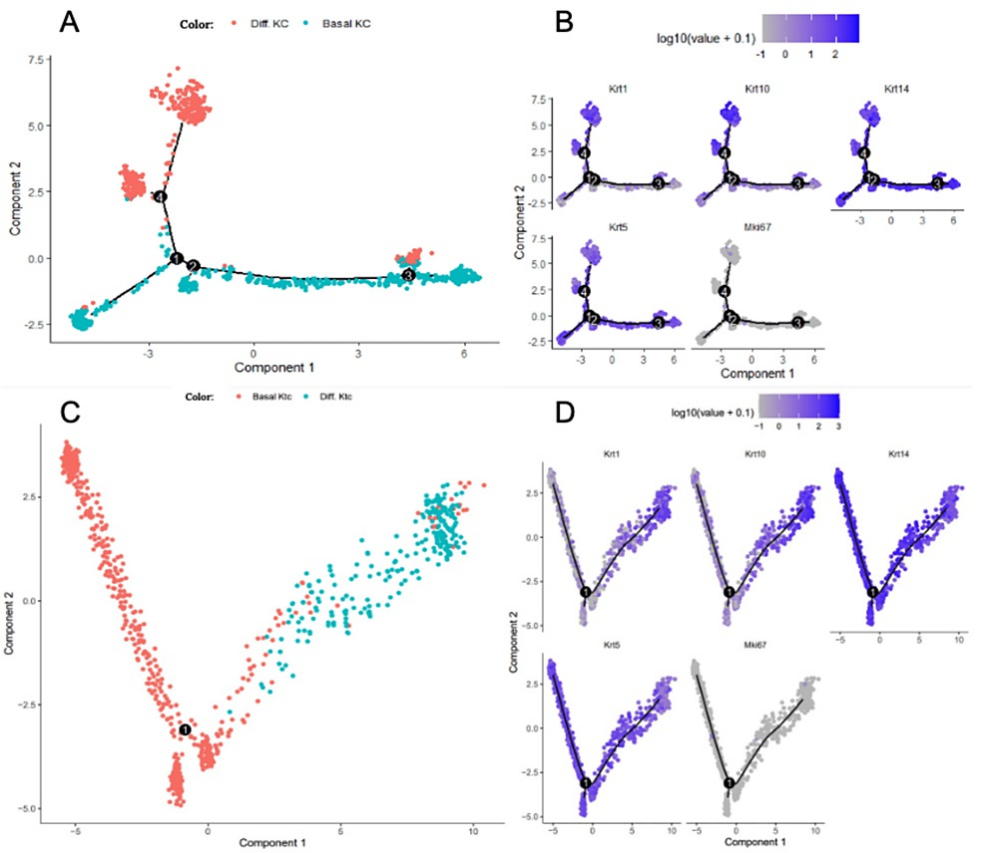
Keratinocyte Differentiation Patterns of Murine Cells From Control and Psoriatic Groups (A) Monocle trajectory of control murine cells. (B) Monocle trajectory of control murine cell gene expression markers. (C) Monocle trajectory of 1% IMQ-applied murine cells. (D) Monocle trajectory of 1% IMQ-applied murine cell gene expression markers IMQ, imiquimod; KC, keratinocyte cell

Monocle trajectories from the control murine cells reveal a nonlinear growth pattern from basal keratinocytes to differentiated keratinocytes, as shown in Figure 2A. In the trajectory, light branching can be observed around four cell states (Figure 2A).

Krt1 and Krt10 can be used as gene markers to track differentiation, which indeed confirms the movement from basal to differentiated keratinocytes in control cells (Figure 2B). Krt5 is also a good model that tracks movement from basal to differentiated keratinocytes, specifically from high expression to low expression (Figure 2B). We found that Krt14 and Mki67 were ineffective as gene markers as they were either overexpressed or underexpressed, respectively, from the movement of basal to differentiated keratinocytes (Figure 2B).

Monocle trajectories from the IMQ-induced murine cells reveal a linear growth pattern from basal keratinocytes to differentiated keratinocytes, with no branching observed (Figure 2C).

Again, Krt1 and Krt10 can be used as gene markers to track differentiation, which displays direct keratinocyte differentiation for IMQ cells (Figure 2D). For the IMQ data, we found that Krt14, Mki67, and Krt5 were ineffective as gene markers as they were either overexpressed or underexpressed from the movement of basal to differentiated keratinocytes (Figure 2D). As a result, only Krt1 and Krt10 were the gene markers used to verify differentiation patterns in both control and IMQ cells.

Branch nodes, indicated by the black circles on the monocle trajectories, reveal different outcomes that cells can take. For the control cells, monocle trajectory analysis indicates four possible outcomes for cells. Meanwhile, for the IMQ cells, monocle trajectory analysis indicates only one possible outcome for cells. As a result, IMQ cells are forced to differentiate, as indicated by the singular cell outcome. However, since psoriasis is characterized by the overproliferation of keratinocytes and poor differentiation, it is interesting that the keratinocyte differentiation trajectory of IMQ-treated data is so continuous. Even when mitotic keratinocytes were included, the continuity persisted.

## Discussion

The differences in cell proportions between control and IMQ-induced murine cells confirm psoriasis-like effects. The results from Figure 1E indicate an increase in keratinocyte cells in the IMQ-treated cells compared to the control. This difference most clearly manifested in the basal and differentiated keratinocytes, not the mitotic keratinocytes (which were present in similar proportions in both datasets); this may simply be due to the short duration of six hours after IMQ treatment, after which the IMQ data was collected.

One hallmark of psoriasis is the uncontrolled proliferation and differentiation of keratinocytes [22], which matches our findings. Keratinocytes play an essential role in the progression of psoriasis. Upon stimulation with interleukin (IL) 17 and tumor necrosis factor (TNF)-alpha, keratinocytes release various inflammatory cytokines that create a positive feedback loop toward keratinocyte proliferation [23]. IL-17 is produced with the recruitment of helper T cells, which increases inflammatory responses within the skin [23].

Subsequently, we found that IMQ-induced cells also contained a greater count of immune cells (Figure 1E). In the control dataset, we were not able to distinguish between T cells and monocytes using established marker genes, but we were able to do so in the IMQ dataset. Furthermore, upon integration, it was evident that immune cells were present in a greater proportion in the IMQ dataset than in the control dataset. Both of these findings indicate a greater immune cell presence in the IMQ-treated skin. The increase in the severity and spread of psoriasis correlates with the increase in the number of inflammatory markers [24]. The higher number of immune cells in the IMQ-treated murine model parallels this progression. Keratinocytes are also subject to IL-23 [23], a factor that has been linked to the involvement of T cells, further increasing the presence of the immune response.

Lastly, it was observed that IMQ-induced cells experienced a decrease in hair follicle cells, specifically basal infundibulum, isthmus, and inner root sheath cells (Figure 1E). We noted that inner root sheath cells were entirely absent from the latter dataset. In addition, basal infundibulum cells, as well as outer bulge cells, are present in lower proportions in the IMQ dataset than in the former. Previous research has shown that psoriasis is often accompanied by hair abnormalities and/or hair loss. Particular histological features that have been noted include the thinning of the infundibulum, as well as the destruction of follicular epithelium [25]. Hair damage due to a psoriatic effect in the IMQ-treated skin may account for these cell types being less prominent in the IMQ dataset.

More specifically, IL-33, another cytokine factor recruited in response to inflammation, is associated with the development of psoriatic alopecia, specifically in the scalp [26]. These findings were observed in research conducted by Dai et al., who used IMQ-induced mice and concluded that physiological changes in hair follicle cells mimic that of psoriasis [26].

We also noticed a small proportion of fibroblasts being present in the control dataset. This is possibly due to some dermal contamination, a conclusion consistent with that in the original paper examining the same data.

Looking at the IMQ keratinocytes, we found a smooth pathway transitioning from basal keratinocytes to differentiated keratinocytes. In addition, when looking at gene markers Krt1 and Krt10, the gradual expression of these markers in pseudotime further supports a gradual transition from basal to differentiated keratinocytes [19]. This was an interesting result as psoriasis is characterized by the overproliferation of keratinocytes and poor differentiation [12]. To further confirm our results, mitotic keratinocytes were reintroduced into the dataset. Such results still showed smooth continuity in the differentiation trajectory. A study using the Seq-Well S^3^ single-cell RNA sequencing protocol generated pseudotime trajectory plots of psoriatic keratinocytes that significantly differ from those we generated in our study [27] with a quite abnormal, branched differentiation trajectory. Although there remains some semblance of order from basal to differentiated, to terminally differentiated, there is a significant amount of scattering that was not observed in our results. One explanation for this may be the short duration of the IMQ treatment. Studies using 5% IMQ first observed differentiation abnormalities only after 24 hours [28]. Six hours of 1% IMQ exposure may not have been sufficient to scatter the pseudotime differentiation trajectory in our IMQ-treated keratinocytes. The impact of long-term or repeated IMQ treatment on keratinocyte differentiation trajectory is a topic that requires future investigation.

However, our results match findings from Ma et al., whose monocle trajectory also revealed a linear relationship [29]. It can be hypothesized that because keratinocyte proliferation is uncontrolled, there is less distance to reach termination (Figure 2C) in comparison to the branched route displayed in the monocle trajectory of the control cells (Figure 2A). However, further analysis using new datasets must be performed in order to determine the full viability of IMQ in inducing psoriasis on murine cells.

A few limitations in this study exist. First, all cells that contained greater than 10% mitochondrial expression were removed from the singe-cell RNA sequencing analysis in an attempt to exclude apoptotic cells. However, healthy cells may have been wrongly removed, requiring future research to experiment with the mitochondrial gene expression cap. This reasoning may explain why T cells and monocytes were only found in the control cells, while melanocytes, immune cells, and fibroblasts were only found in IMQ cells. Second, different resolutions can be used to further categorize cell clusters in tSNE plots. Specifically, this data can be reevaluated using a 0.5 resolution, as done by previous research [20,21].

## Conclusions

IMQ-treated murine cells were observed to have increased keratinocyte cell populations in comparison to the control murine cell population, as noted through single-cell RNA sequencing. Additionally, higher immune cell populations were found in IMQ murine cells compared to control murine cells, suggesting its role in mimicking psoriatic symptoms. However, monocle trajectory analysis also revealed a linear differentiation pattern for IMQ-treated cells, raising concerns about the efficacy of IMQ to mirror psoriatic conditions as psoriasis is characterized by uncontrolled keratinocyte differentiation.

Further research using other datasets and human study validation of this model is needed to improve our understanding and pinpoint potential therapeutic targets. We also need to clarify the full spectrum of IMQ effects and advance our knowledge of the disease’s pathophysiology for treatment development.
